# Supplementary figures and images for: Elucidating the patterns of pleiotropy and its biological relevance in maize
Source: PLoS Genet. 2023 Mar 21;19(3):e1010664. doi: 10.1371/journal.pgen.1010664 (PMC10030035; doi:10.1371/journal.pgen.1010664)

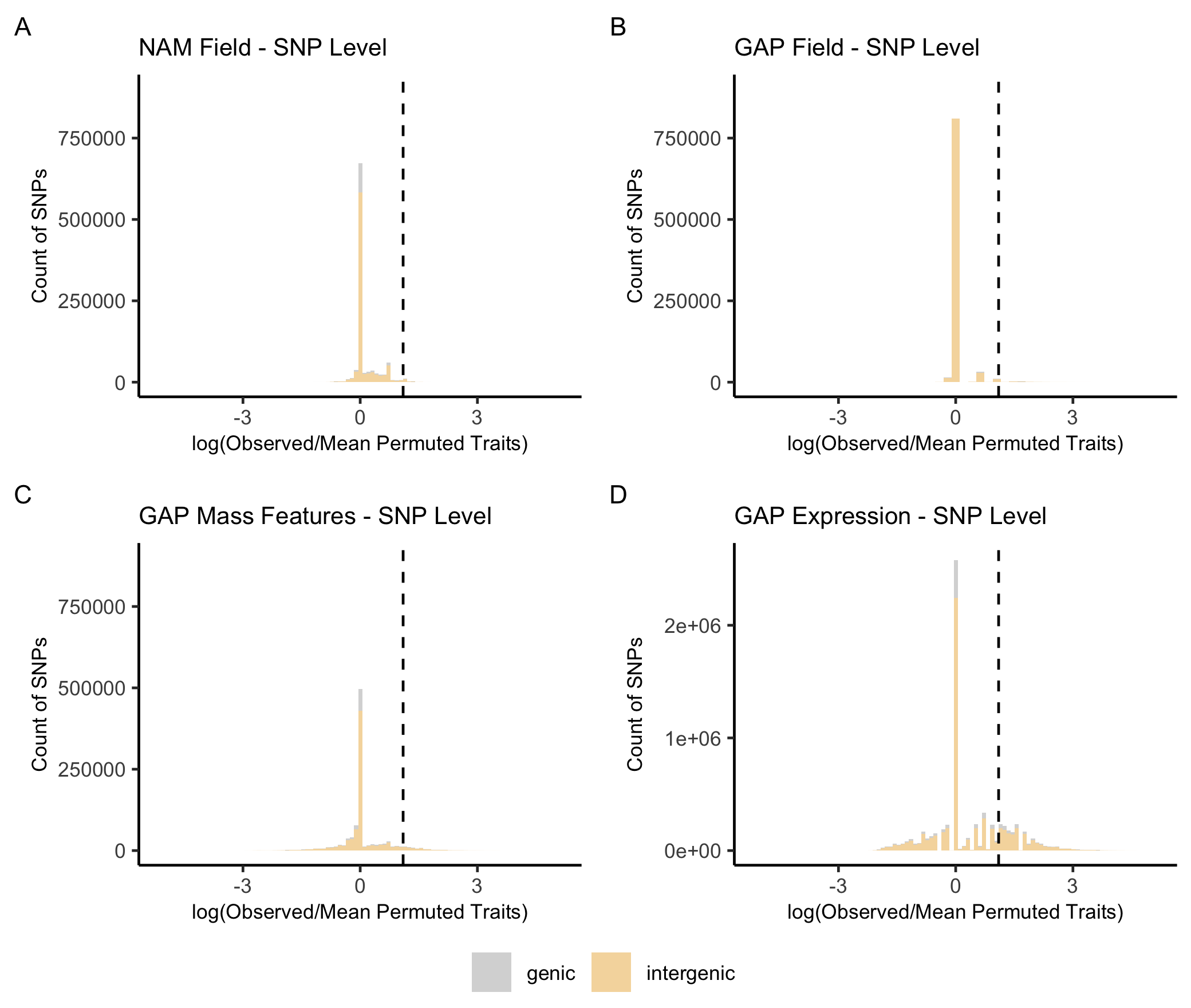

Supplement: S1 Fig — Of the SNPs showing a five-fold higher proportion of pleiotropy over their permutations (right of the vertical dashed line). Each value along the x-axis was calculated from the natural log of the number of observed traits mapping to each SNP divided by the mean count of traits in the permuted data with a pseudo-count of plus one in the numerator and denominator. Values left of the vertical dashed line indicate higher pleiotropy in the permuted data versus the observed data suggesting the prevalence of high noise or no trait-SNP associations in either the observed or permuted data (peak at zero). Distributions are split into genic (gray) and intergenic (yellow) SNPs for (a) NAM field, (b) GAP field, (c) GAP mass features, and (d) GAP expression traits. (TIF) [file pgen.1010664.s010.tif]

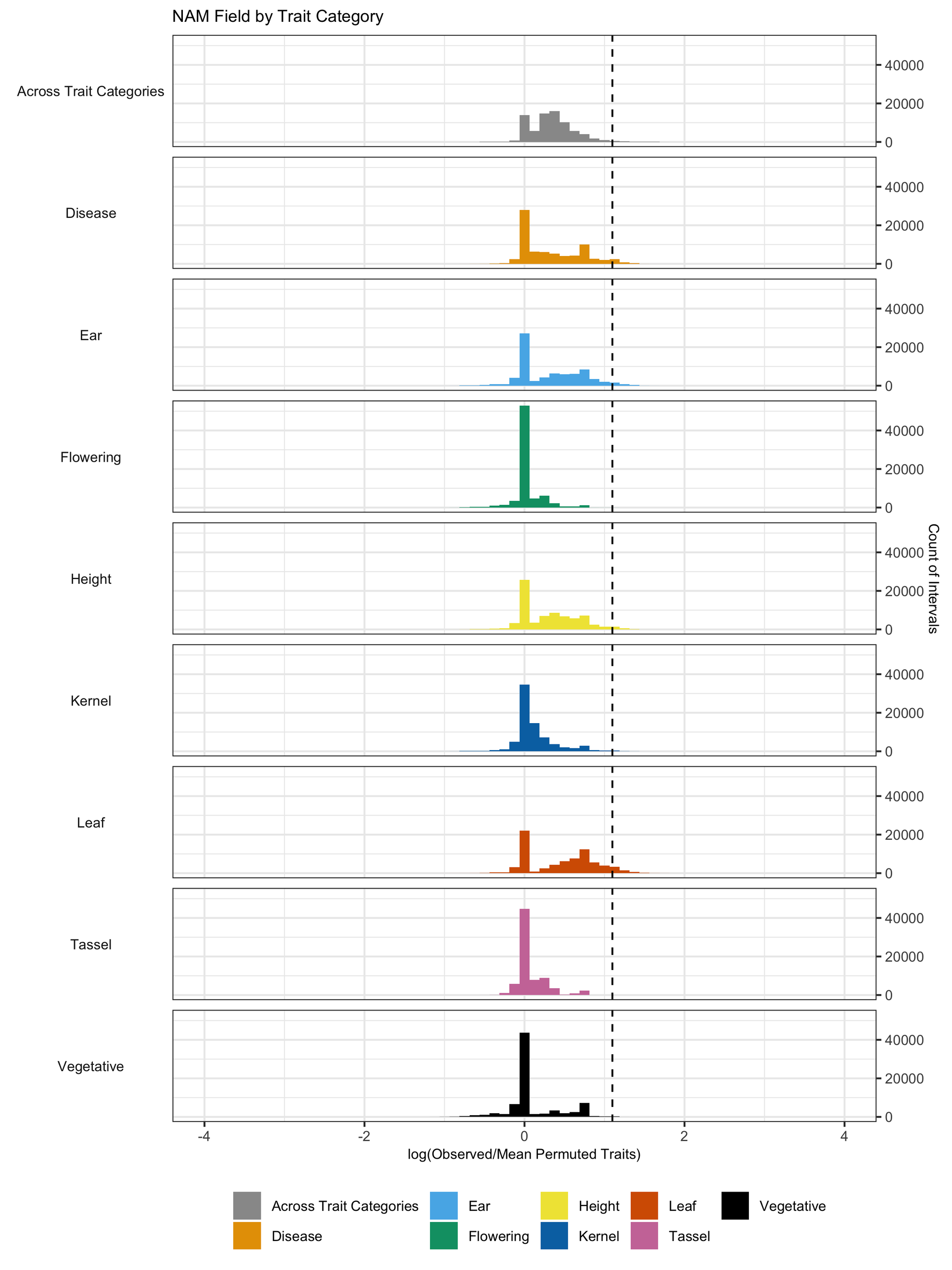

Supplement: S2 Fig — Very few intervals show a five-fold higher proportion of pleiotropy over their permutations (right of the vertical dashed line) in the NAM field traits. Each value along the x-axis was calculated within a trait category from the natural log of the number of observed traits mapping to each interval divided by the mean count of traits in the permuted data with a pseudo-count of plus one in the numerator and denominator. Values left of the vertical dashed line indicate higher pleiotropy in the permuted data versus the observed data suggesting the prevalence of high noise or no trait-SNP associations in either the observed or permuted data (peak at zero). (TIF) [file pgen.1010664.s011.tif]

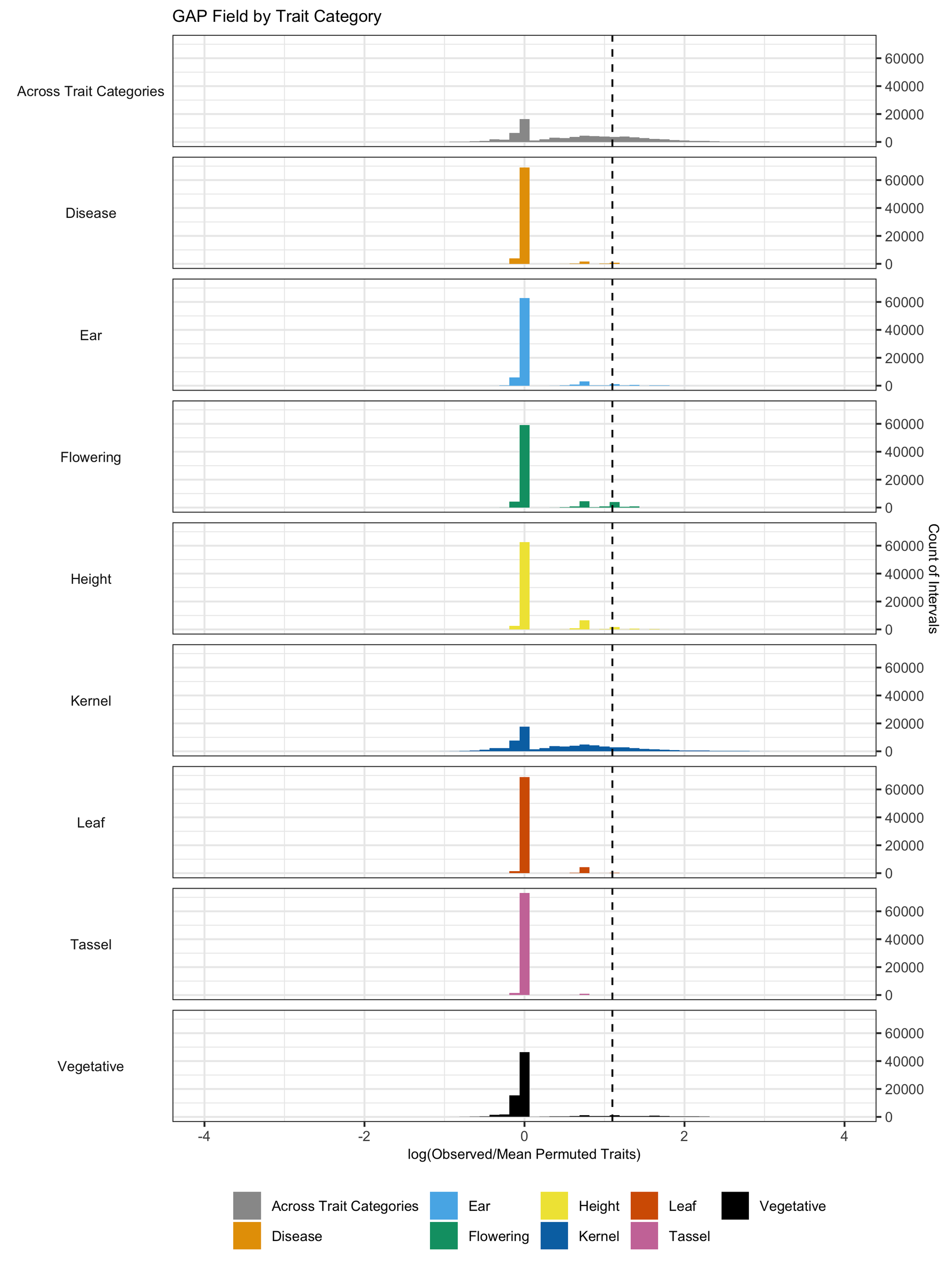

Supplement: S3 Fig — Very few intervals show a five-fold higher proportion of pleiotropy over their permutations (right of the vertical dashed line) in the GAP field traits. Each value along the x-axis was calculated within a trait category from the natural log of the number of observed traits mapping to each interval divided by the mean count of traits in the permuted data with a pseudo-count of plus one in the numerator and denominator. Values left of the vertical dashed line indicate higher pleiotropy in the permuted data versus the observed data suggesting the prevalence of high noise or no trait-SNP associations in either the observed or permuted data (peak at zero). (TIF) [file pgen.1010664.s012.tif]

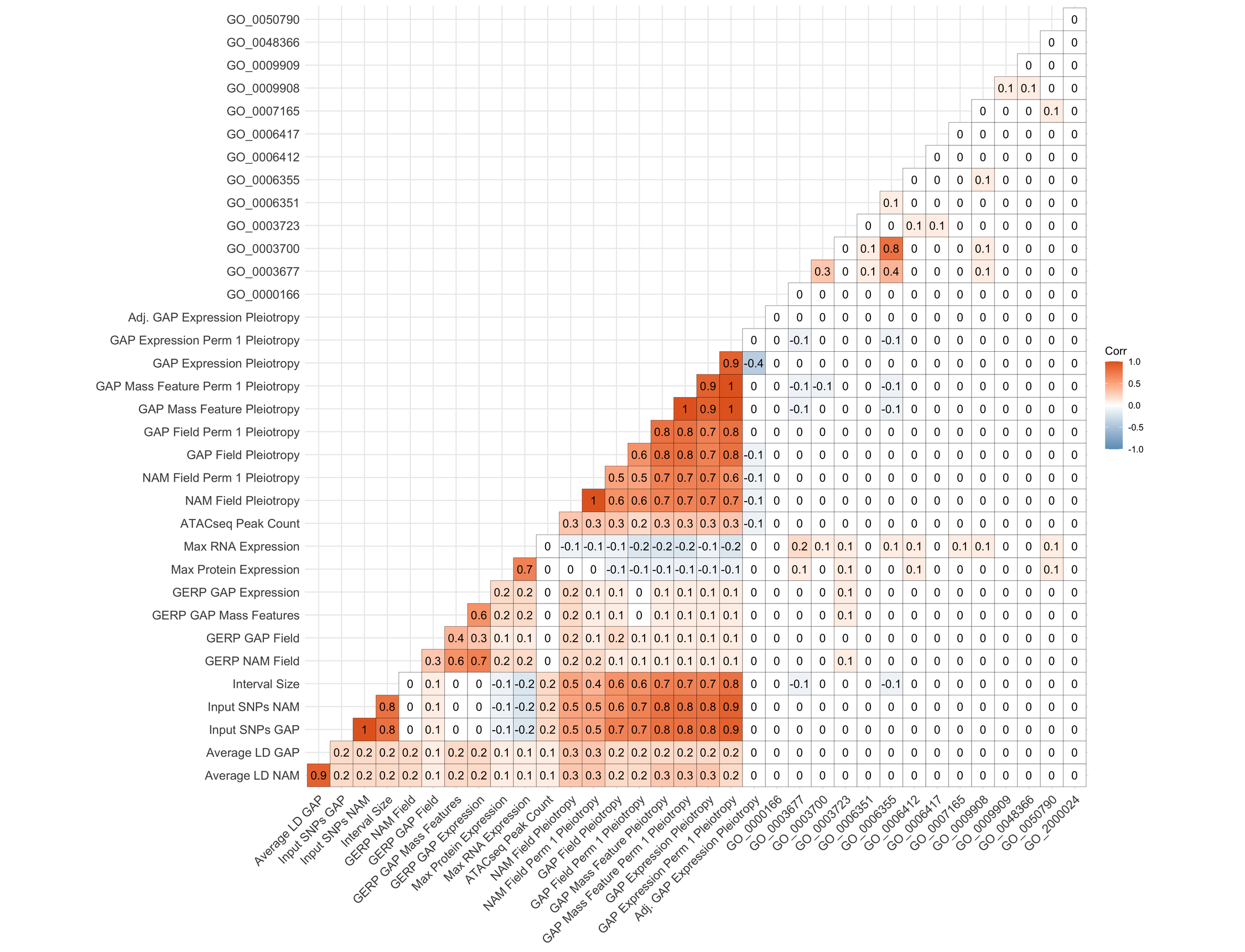

Supplement: S4 Fig — Only the first permuted value from each of the four population-trait categories was included in the correlation matrix for simplicity. (TIF) [file pgen.1010664.s013.tif]

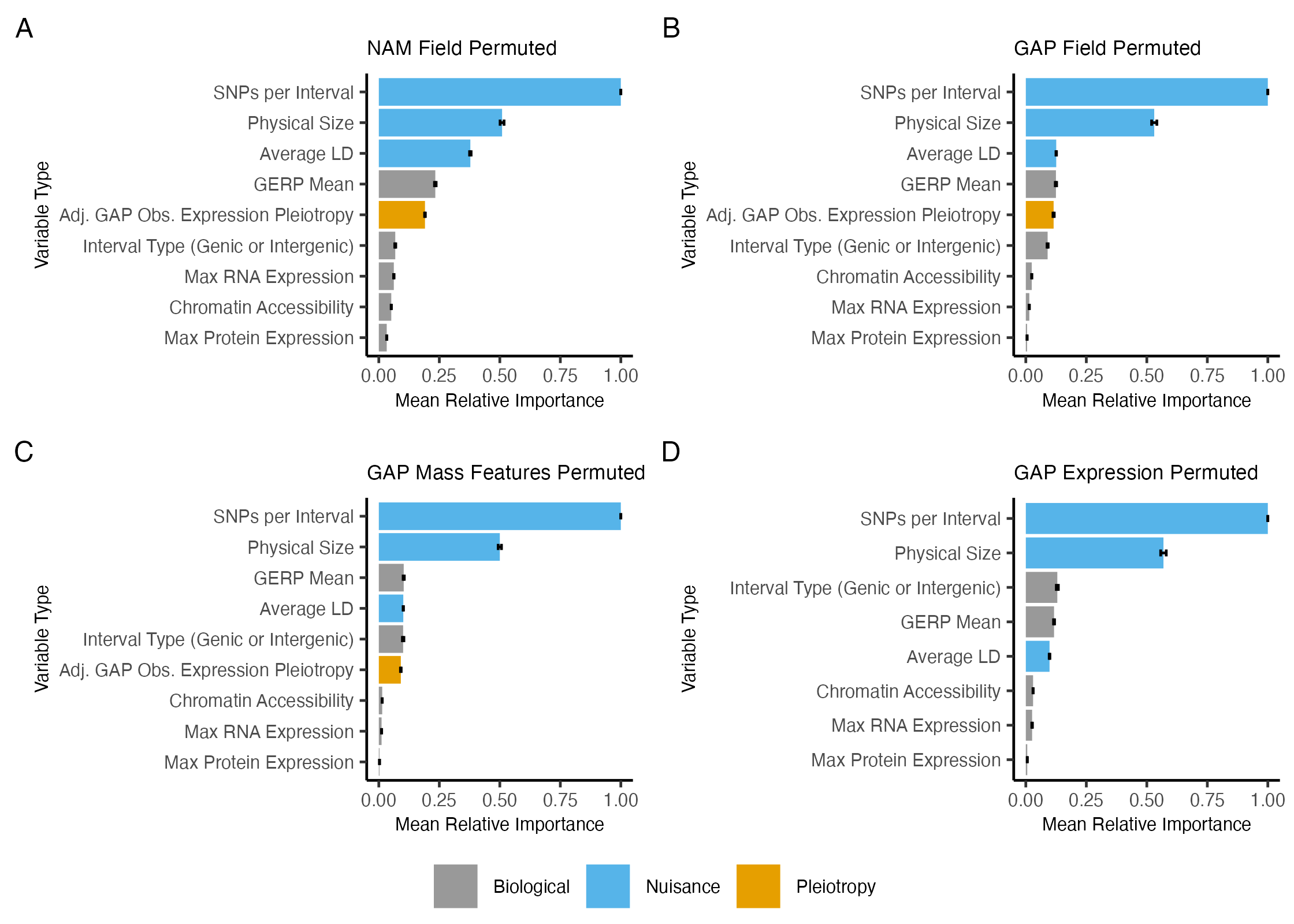

Supplement: S5 Fig — Across all four population-trait categories, nuisance variables showed higher relative importance over biological features. The plots show the observed data for the (a) NAM field, (b) GAP field, (c) GAP mass features, and (d) GAP expression data. The bar charts depict the mean relative importance and standard error of each variable from a leave-one-chromosome-out model. (TIF) [file pgen.1010664.s014.tif]

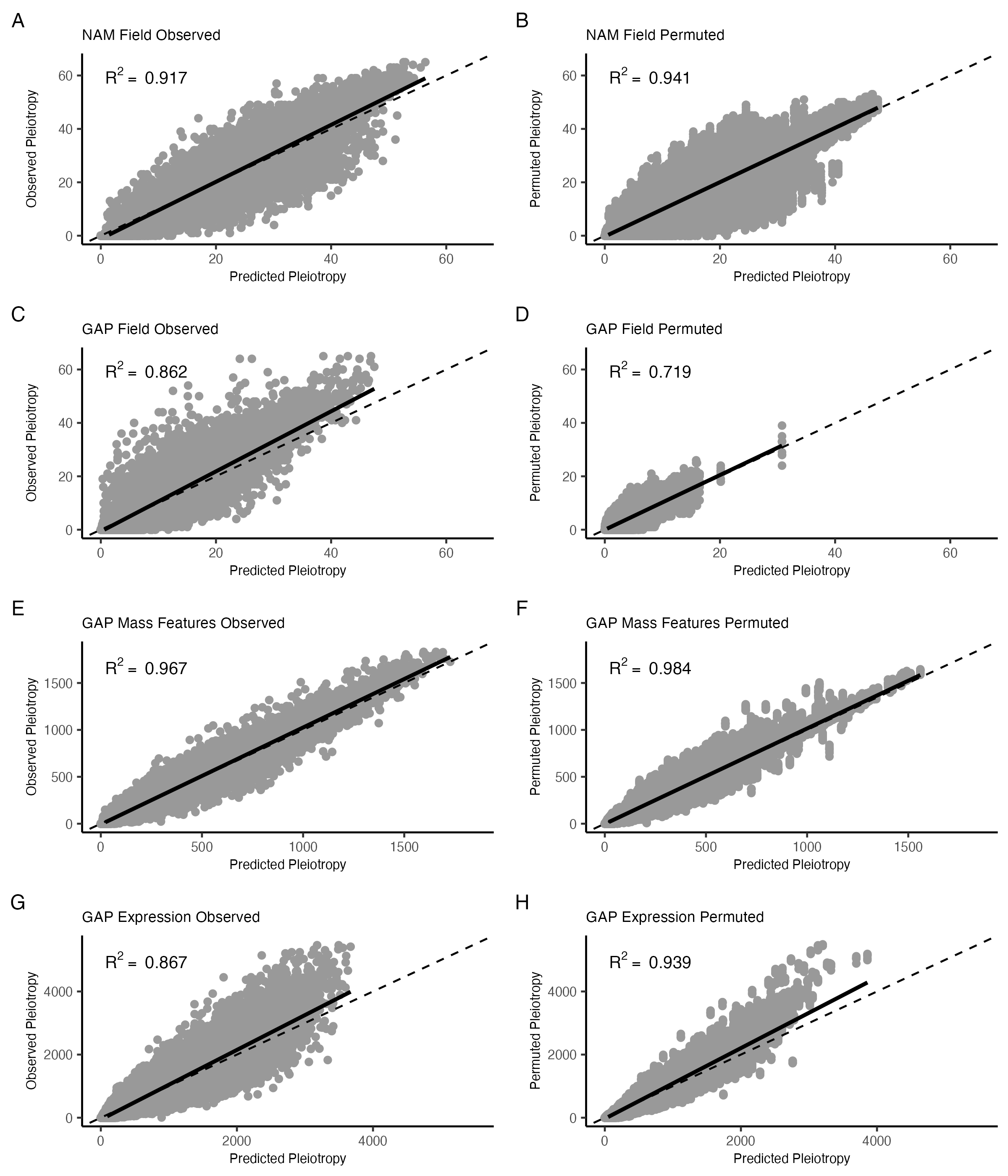

Supplement: S6 Fig — The dashed line represents the 1–1 identity line, while the solid line represents fitted values. Panels (a), (c), (e), and (g) show the observed results while panels (b), (d), (f), and (h) show the permuted results. Panels (a) and (b) show NAM field, (c) and (d) GAP field, (e) and (f) GAP mass features, and (g) and (h) GAP expression. The plots show the observed and predicted values across all held-out chromosomes from the leave-one-chromosome-out model. (TIF) [file pgen.1010664.s015.tif]

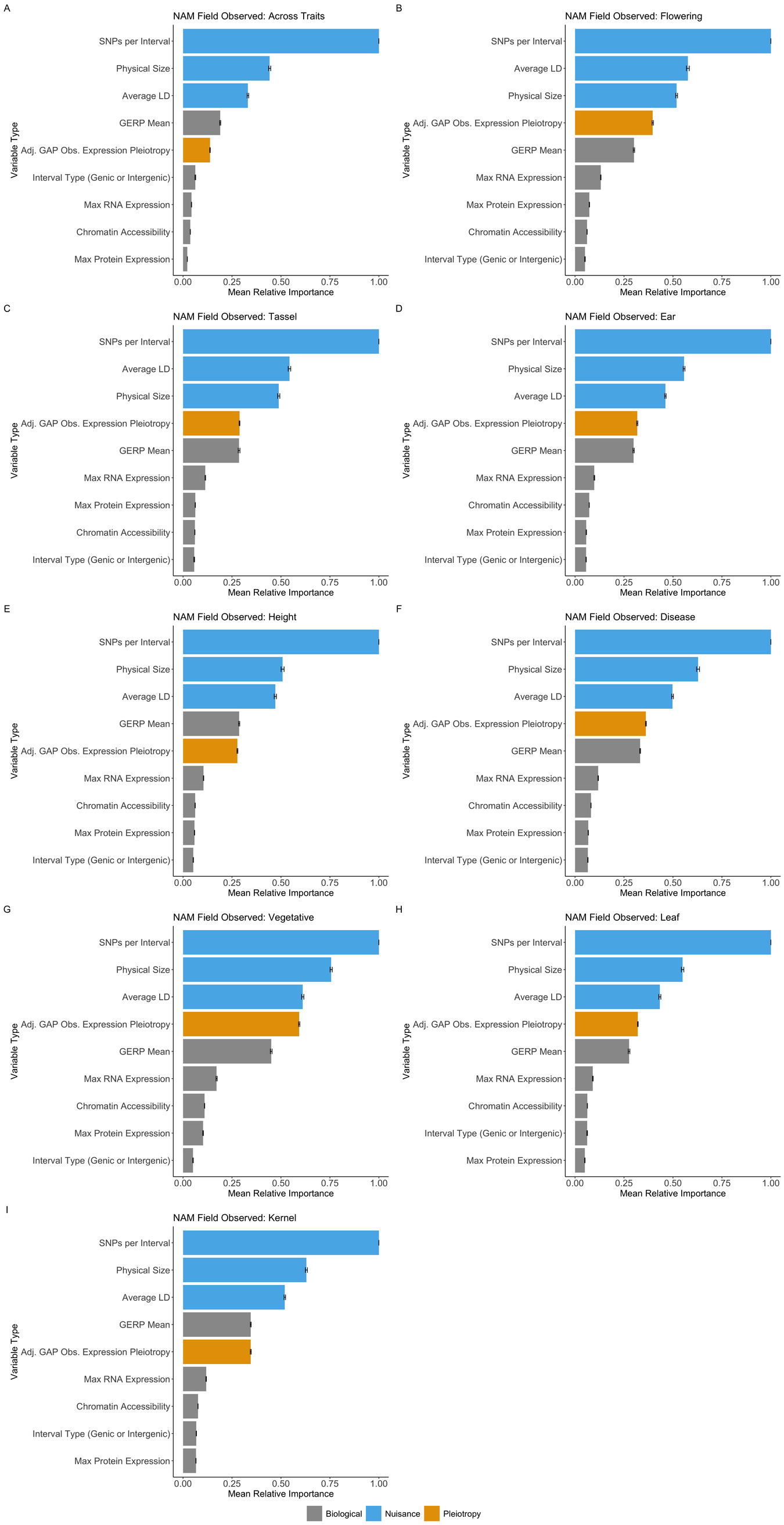

Supplement: S7 Fig — Across the eight trait types, nuisance variables showed higher relative importance over biological features. The plots show the observed data for the NAM field results (a) across all traits, (b) flowering, (c) tassel, (d) ear, (e) height, (f) disease, (g) vegetative, (h) leaf, and (i) kernel traits. The bar charts depict the mean relative importance and standard error of each variable from a leave-one-chromosome-out model. (TIF) [file pgen.1010664.s016.tif]

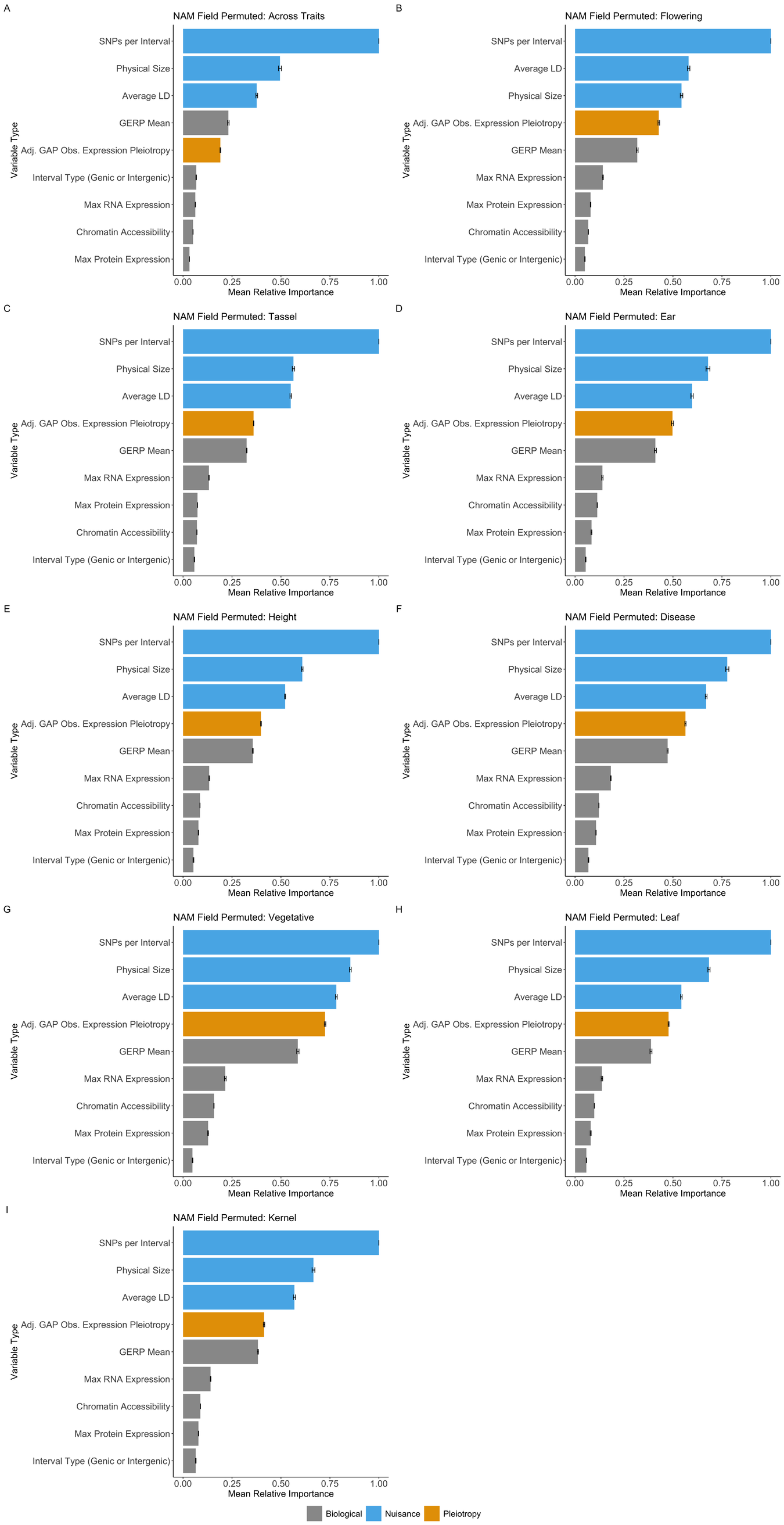

Supplement: S8 Fig — Across the eight trait types, nuisance variables showed higher relative importance over biological features. The plots show the permuted data for the NAM field results (a) across all traits, (b) flowering, (c) tassel, (d) ear, (e) height, (f) disease, (g) vegetative, (h) leaf, and (i) kernel traits. The bar charts depict the mean relative importance and standard error of each variable from a leave-one-chromosome-out model. (TIF) [file pgen.1010664.s017.tif]

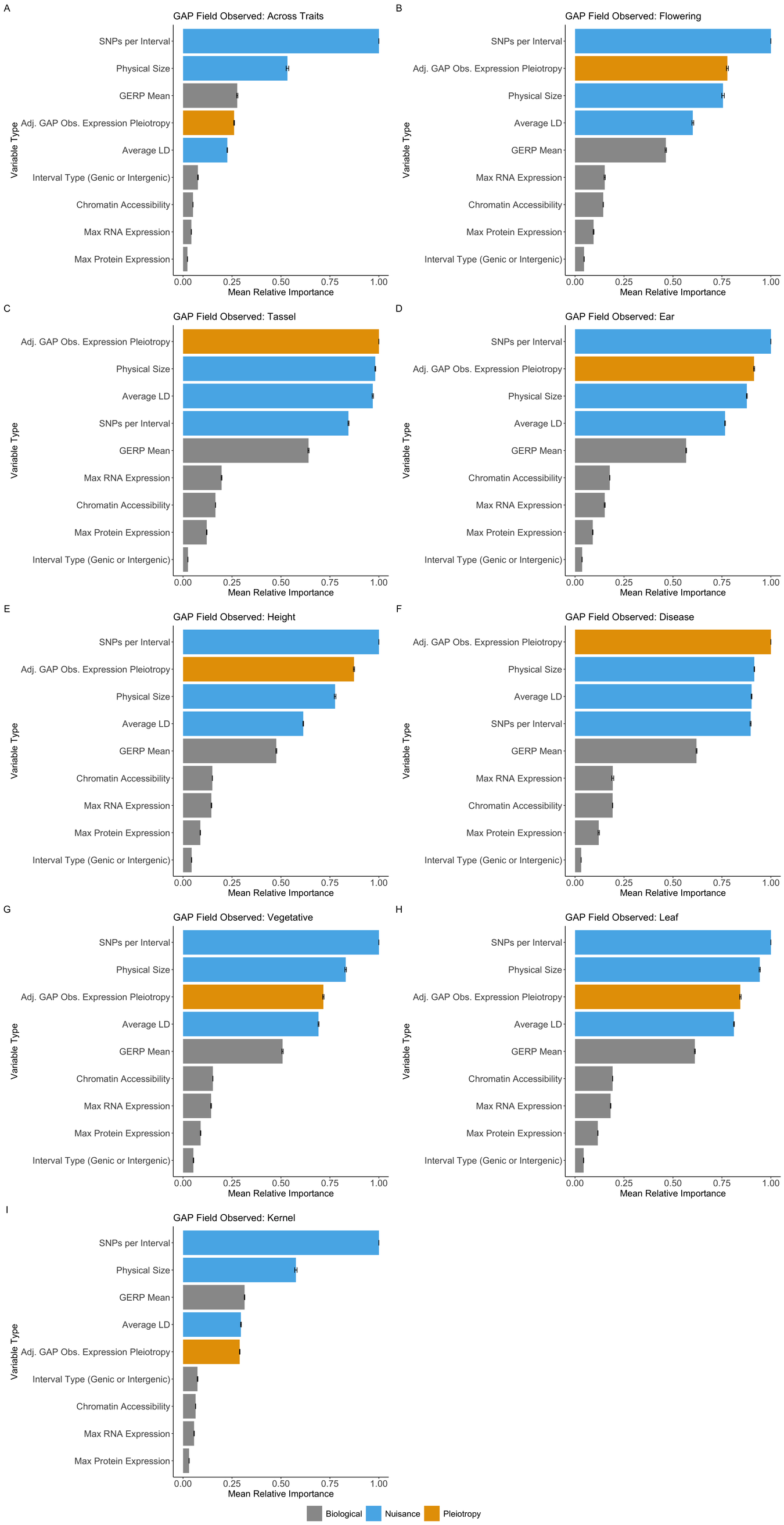

Supplement: S9 Fig — Across the eight trait types, nuisance variables showed higher relative importance over biological features. The plots show the observed data for the GAP field results (a) across all traits, (b) flowering, (c) tassel, (d) ear, (e) height, (f) disease, (g) vegetative, (h) leaf, and (i) kernel traits. The bar charts depict the mean relative importance and standard error of each variable from a leave-one-chromosome-out model. (TIF) [file pgen.1010664.s018.tif]

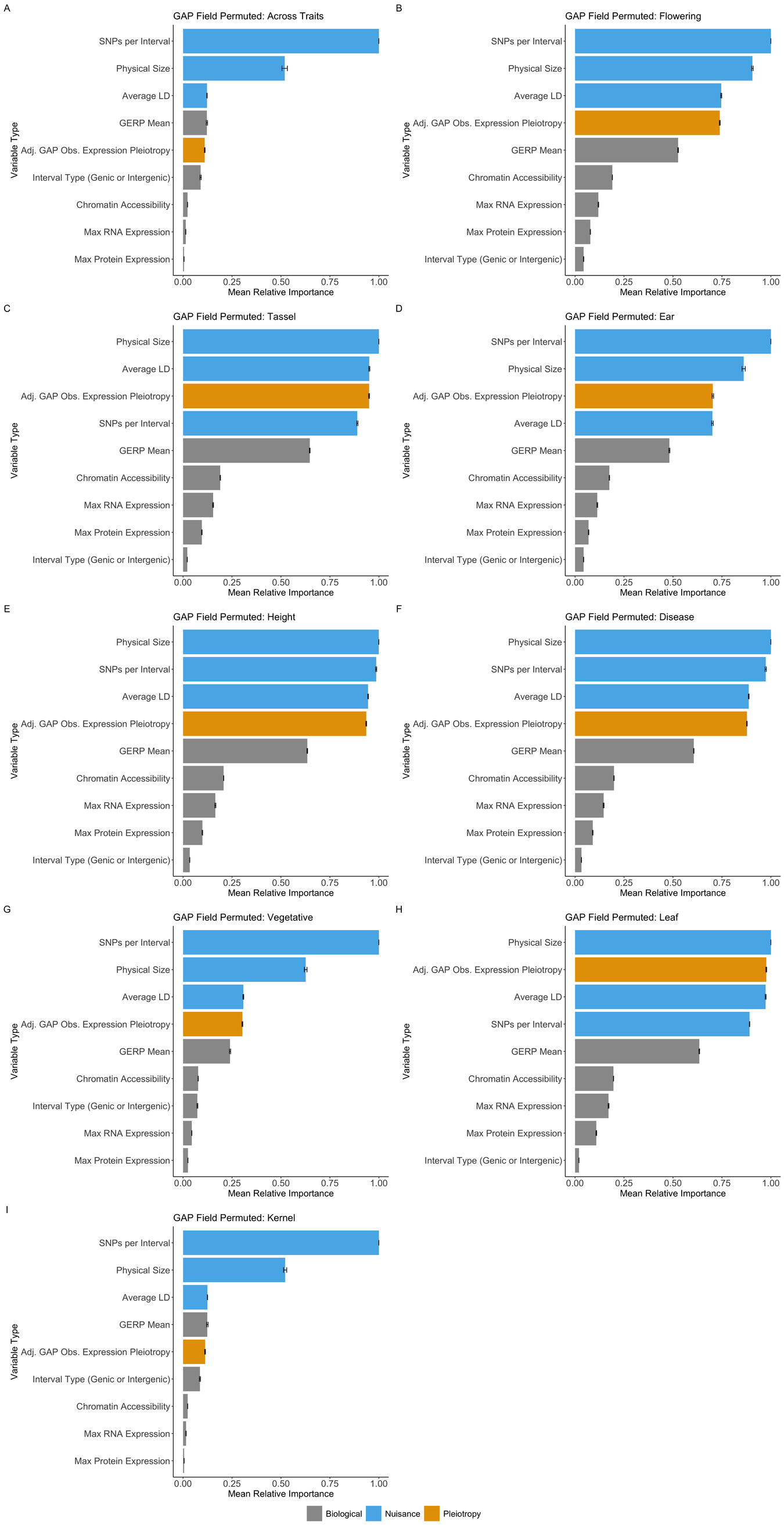

Supplement: S10 Fig — Across the eight trait types, nuisance variables showed higher relative importance over biological features. The plots show the data for the GAP field results (a) across all traits, (b) flowering, (c) tassel, (d) ear, (e) height, (f) disease, (g) vegetative, (h) leaf, and (i) kernel traits. The bar charts depict the mean relative importance and standard error of each variable from a leave-one-chromosome-out model. (TIF) [file pgen.1010664.s019.tif]

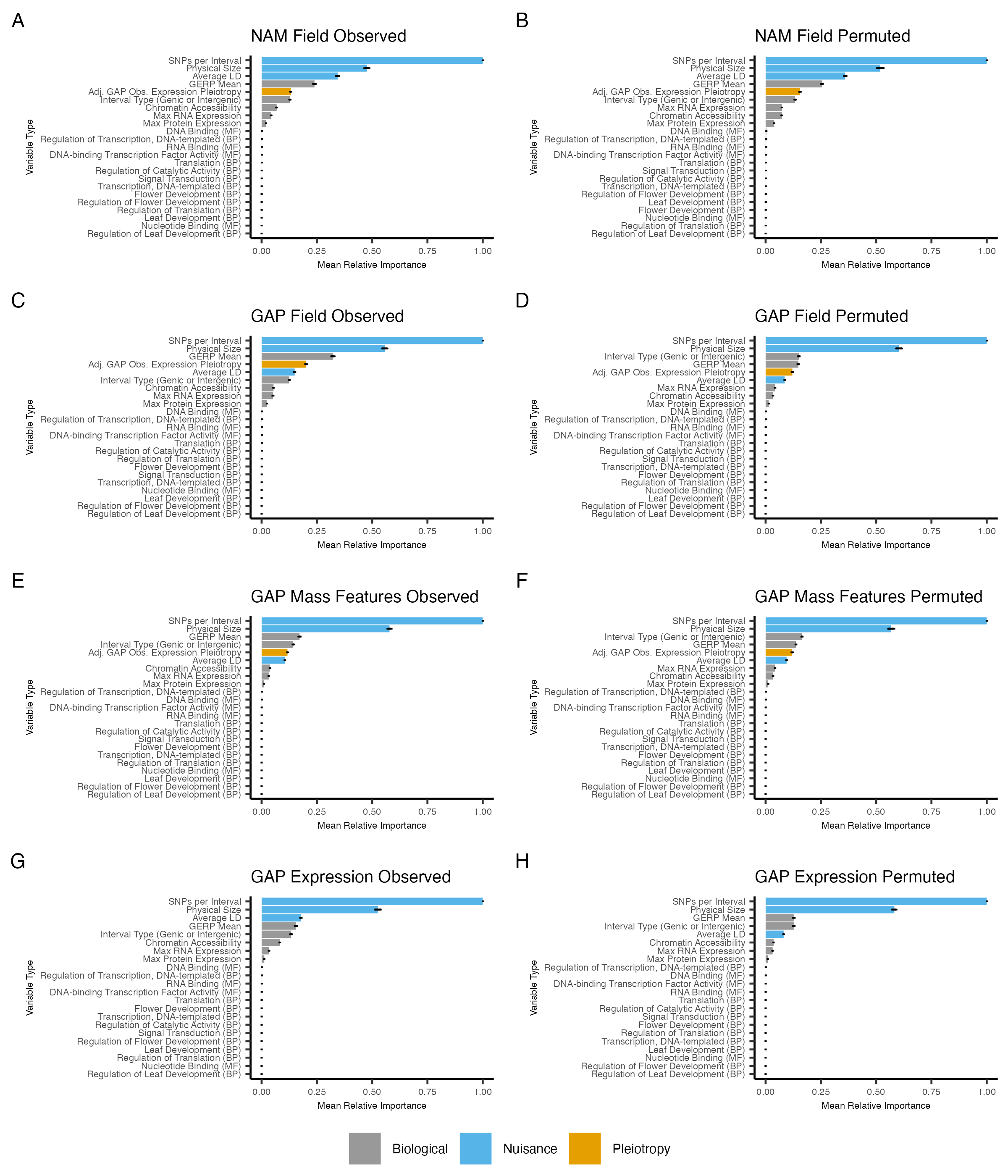

Supplement: S11 Fig — Across all four population-trait categories, nuisance variables showed higher relative importance over biological features. The plots show data for the observed data in panels (a), (c), (e), and (g) and the permuted data in panels (b), (d), (f), and (h). Panels (a) and (b) show NAM field results, (c) and (d) GAP field, (e) and (f) GAP mass features, and (g) and (h) GAP expression data. The bar charts depict the mean relative importance and standard error of each variable from a leave-one-chromosome-out model. (TIF) [file pgen.1010664.s020.tif]

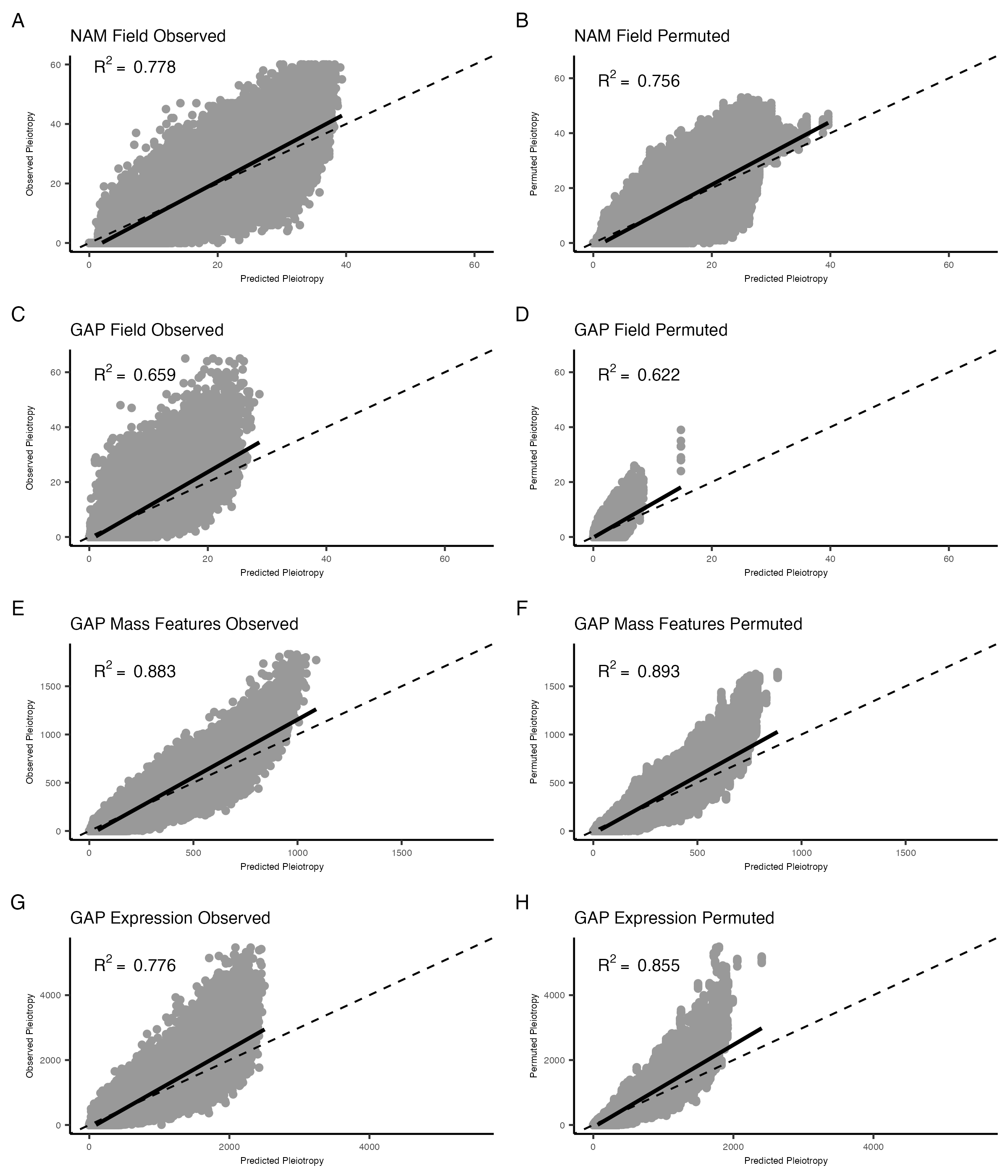

Supplement: S12 Fig — The dashed line represents the 1–1 identity line, while the solid line represents fitted values. Panels (a), (c), (e), and (g) show the observed results while panels (b), (d), (f), and (h) show the permuted results. Panels (a) and (b) show NAM field, (c) and (d) GAP field, (e) and (f) GAP mass features, and (g) and (h) GAP expression. The plots show the observed and predicted values across all held-out chromosomes from the leave-one-chromosome-out model. (TIF) [file pgen.1010664.s021.tif]

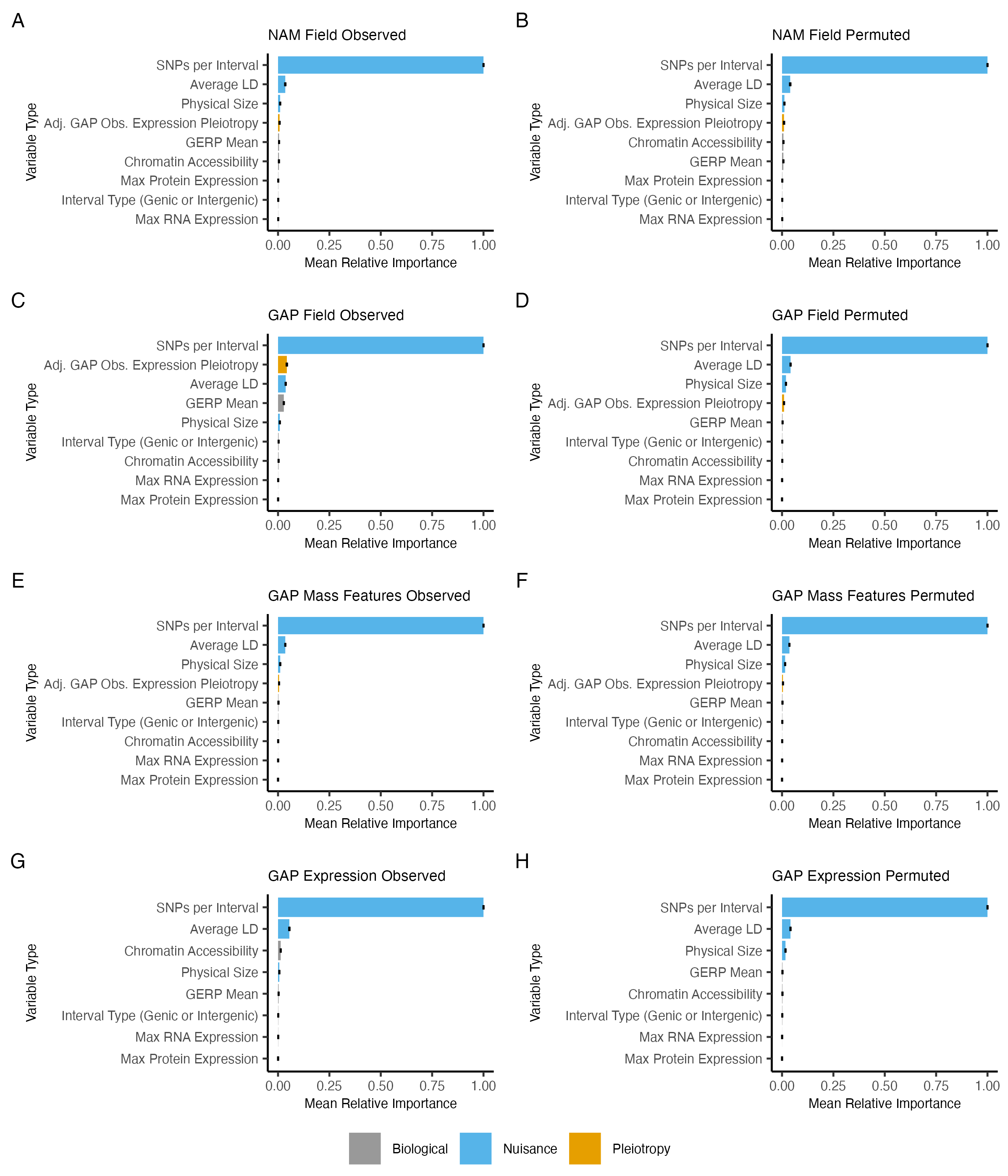

Supplement: S13 Fig — Across all four population-trait categories, nuisance variables results showed higher relative importance over biological features. The plots show data for the observed data in panels (a), (c), (e), and (g) and the permuted data in panels (b), (d), (f), and (h). Panels (a) and (b) show NAM field results, (c) and (d) GAP field, (e) and (f) GAP mass features, and (g) and (h) GAP expression data. The bar charts depict the mean relative importance and standard error of each variable from a leave-one-chromosome-out model. (TIF) [file pgen.1010664.s022.tif]

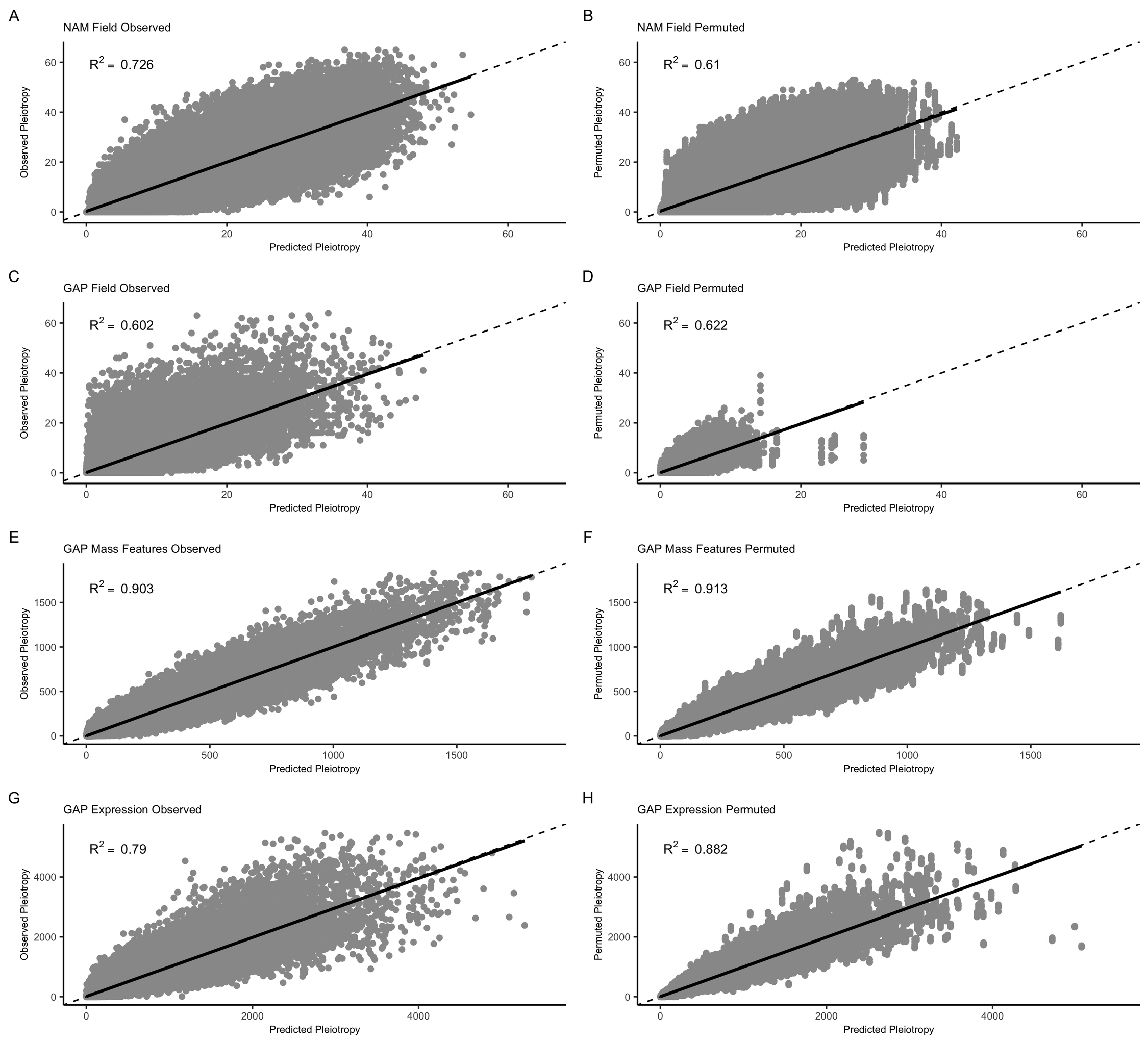

Supplement: S14 Fig — The dashed line represents the 1–1 identity line, while the solid line represents fitted values. Panels (a), (c), (e), and (g) show the observed results while panels (b), (d), (f), and (h) show the permuted results. Panels (a) and (b) show NAM field, (c) and (d) GAP field, (e) and (f) GAP mass features, and (g) and (h) GAP expression. The plots show the observed and predicted values across all held-out chromosomes from the leave-one-chromosome-out model. (TIF) [file pgen.1010664.s023.tif]

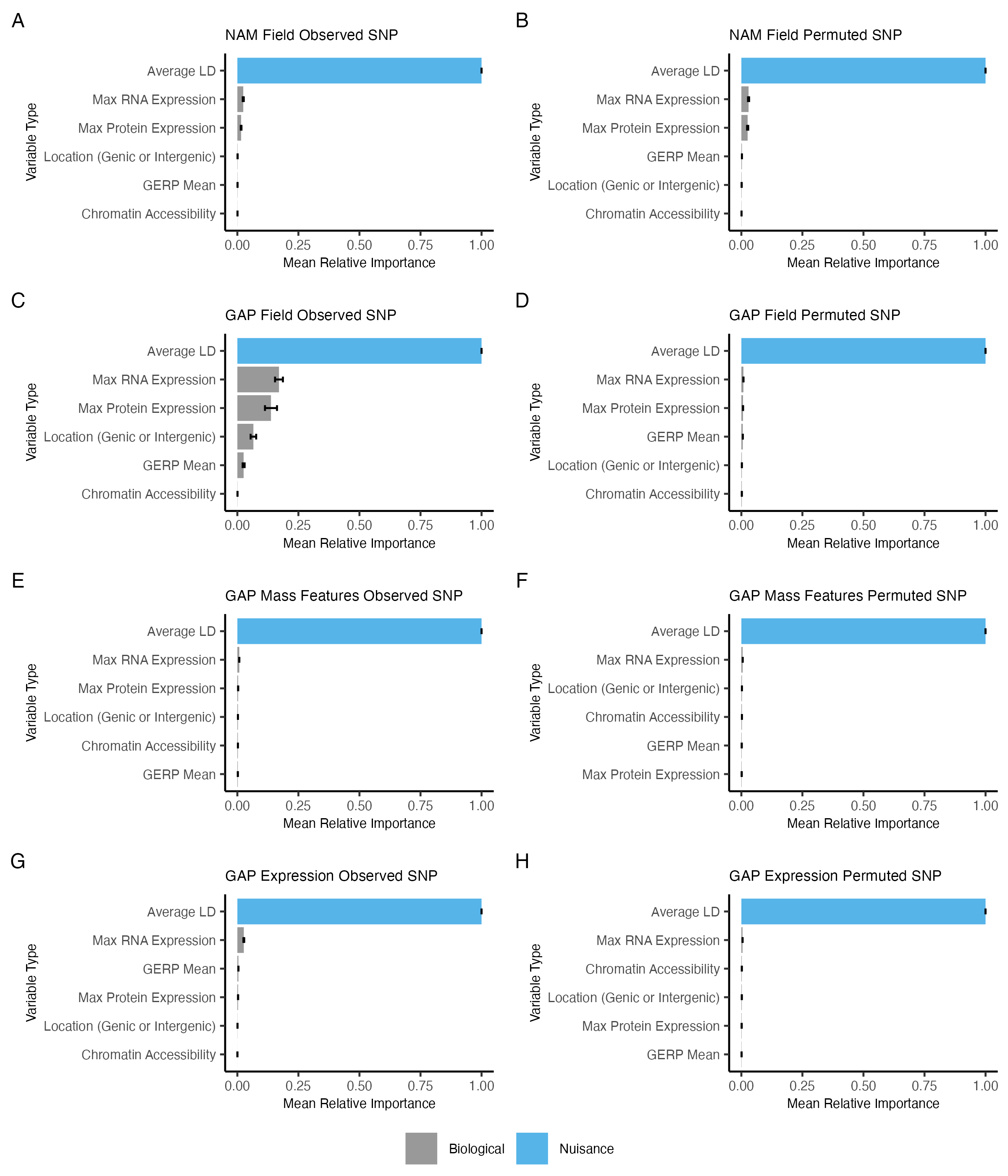

Supplement: S15 Fig — Across all four population-trait categories, nuisance variables showed higher relative importance over biological features. The plots show data for the observed data in panels (a), (c), (e), and (g) and the permuted data in panels (b), (d), (f), and (h). Panels (a) and (b) show NAM field results, (c) and (d) GAP field, (e) and (f) GAP mass features, and (g) and (h) GAP expression data. The bar charts depict the mean relative importance and standard error of each variable from a leave-one-chromosome-out model. (TIF) [file pgen.1010664.s024.tif]

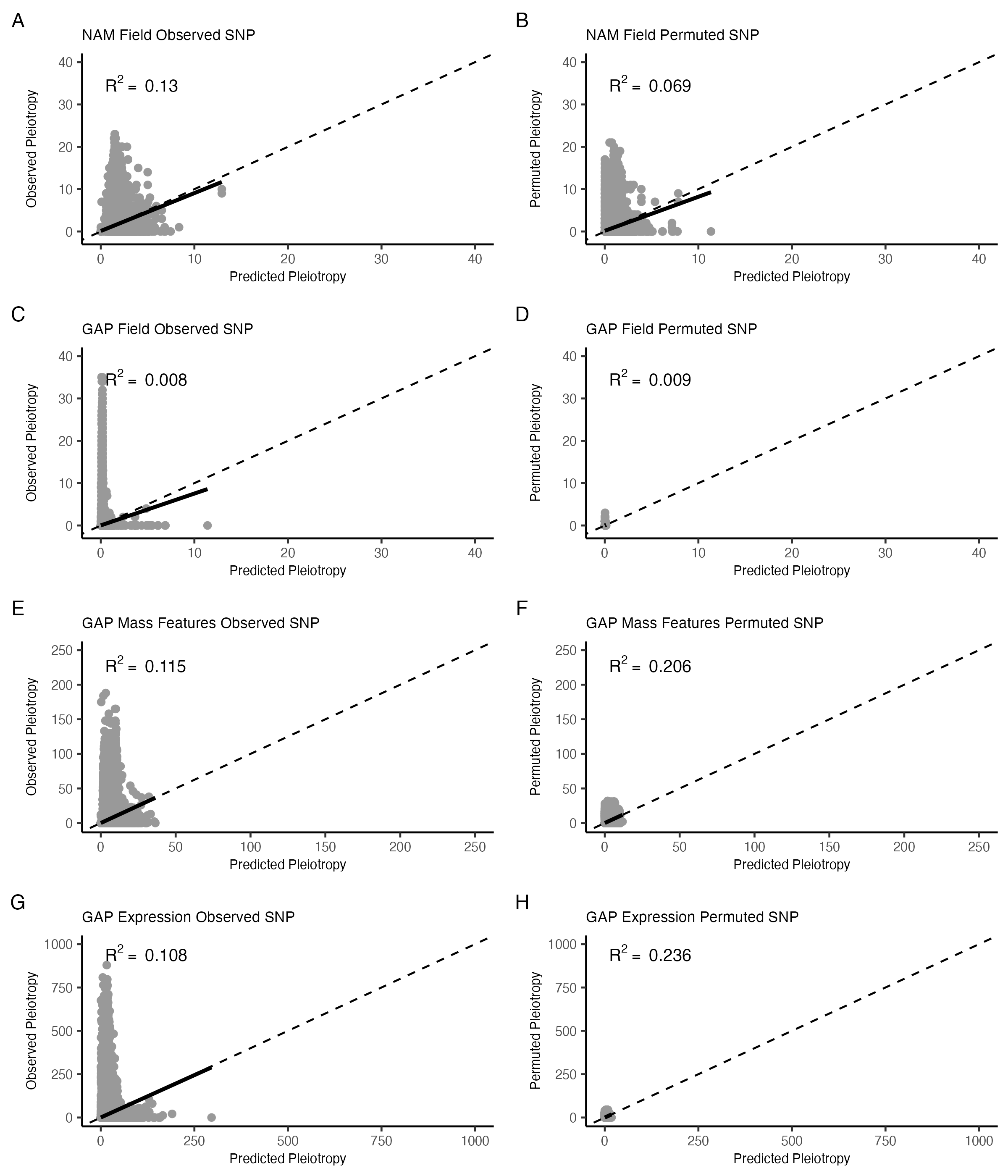

Supplement: S16 Fig — The dashed line represents the 1–1 identity line, while the solid line represents fitted values. Panels (a), (c), (e), and (g) show the observed results while panels (b), (d), (f), and (h) show the permuted results. Panels (a) and (b) show NAM field, (c) and (d) GAP field, (e) and (f) GAP mass features, and (g) and (h) GAP expression. The plots show the observed and predicted values across all held-out chromosomes from the leave-one-chromosome-out model. (TIF) [file pgen.1010664.s025.tif]

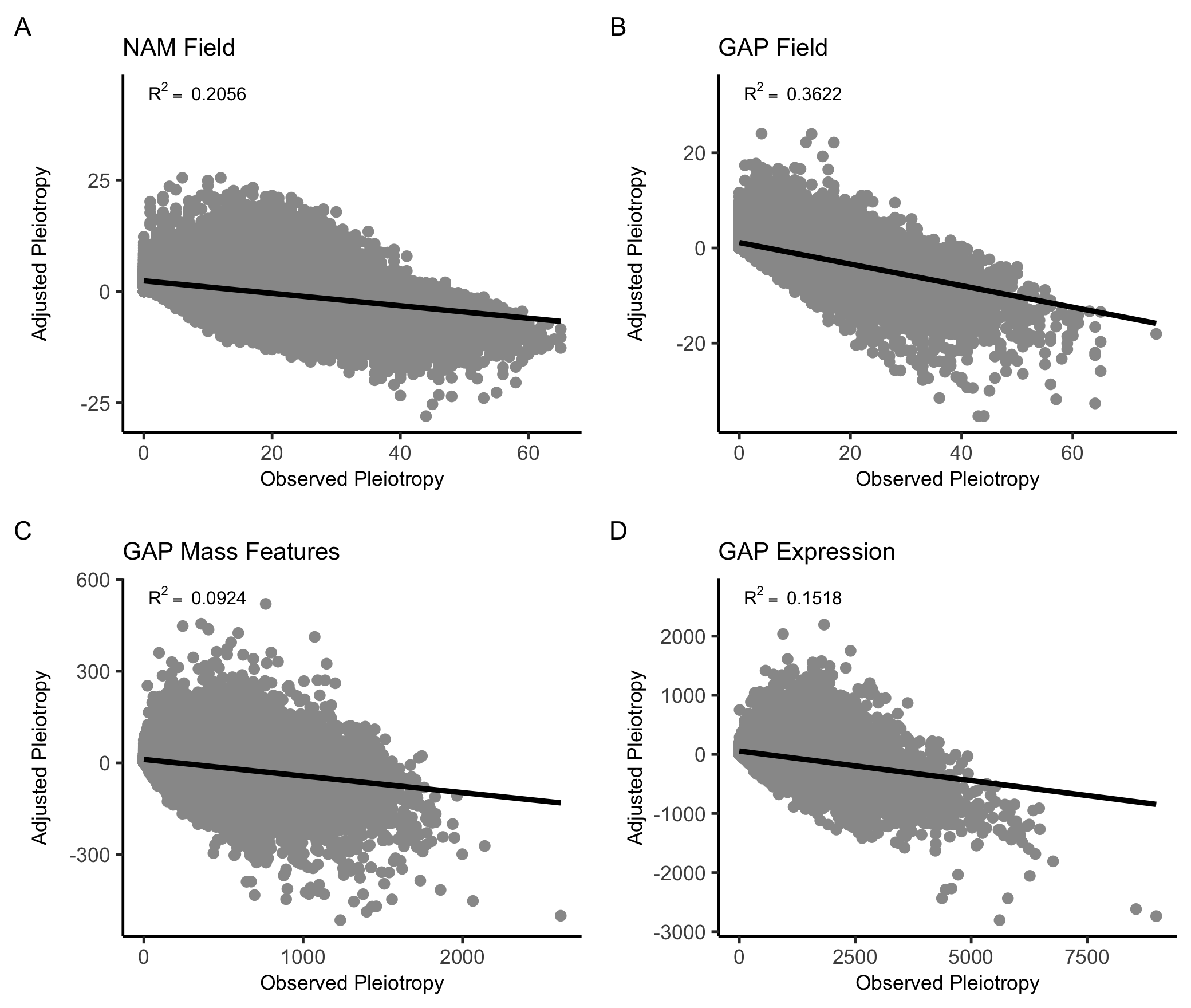

Supplement: S17 Fig — The scatter plots show the relationship between the adjusted and observed pleiotropy values for (a) NAM field, (b) GAP field, (c) GAP mass features, and (d) GAP expression traits. Values in the top right of each plot show the R2 values from correlating the adjusted versus unadjusted pleiotropy data. (TIF) [file pgen.1010664.s026.tif]

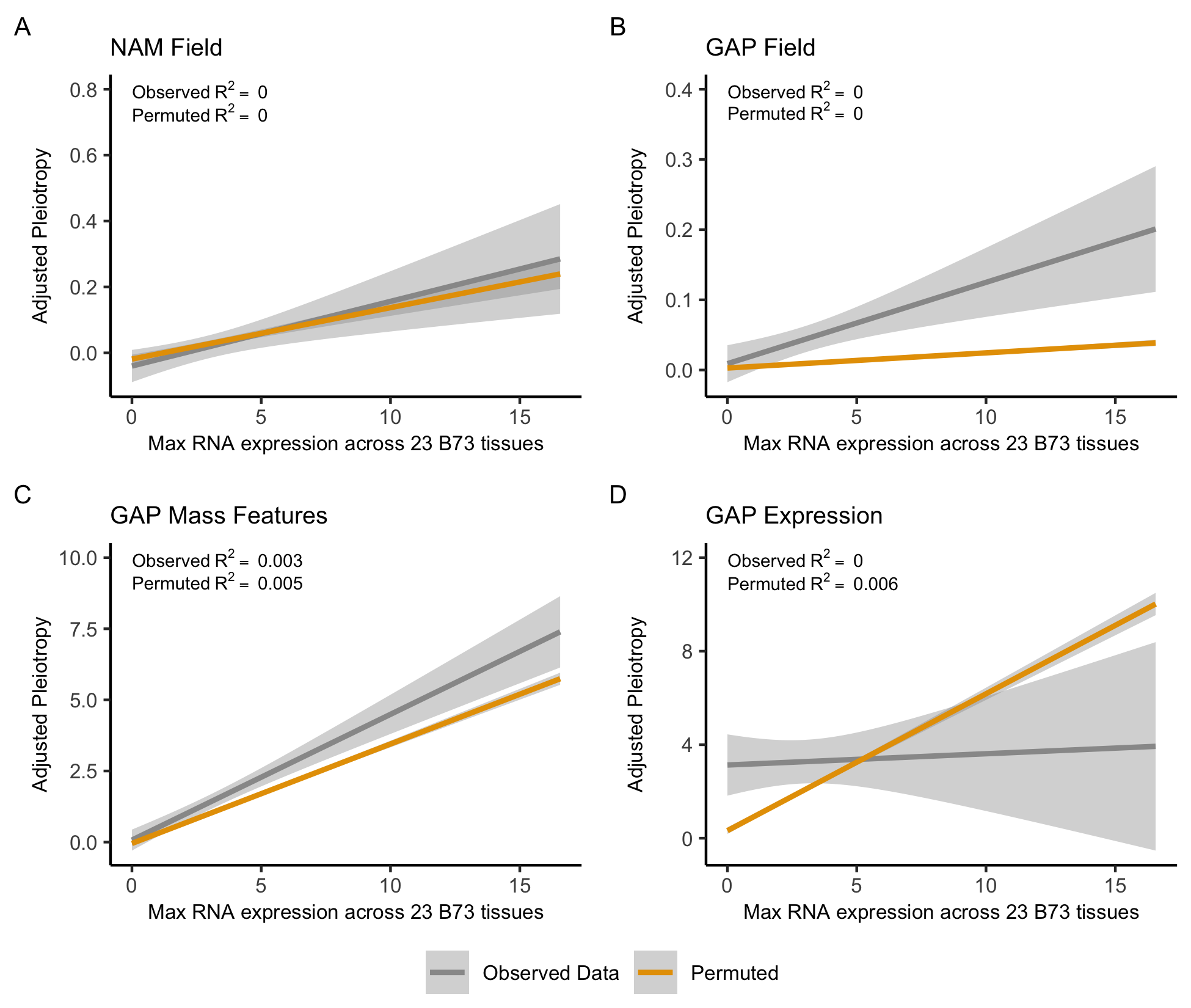

Supplement: S18 Fig — The line plots of adjusted pleiotropy for only genic ranges against the max RNA expression show the observed (gray lines) and permuted data (yellow lines). Panels show adjusted pleiotropy scores for (a) NAM field, (b) GAP field, (c) GAP mass features, and (d) GAP expression traits. Values in the top right of each plot show the R2 values from correlating the observed genic and permuted genic adjusted pleiotropy data separately against the max RNA expression value across 23 B73 tissues. Shading around lines shows the 95% confidence interval. (TIF) [file pgen.1010664.s027.tif]

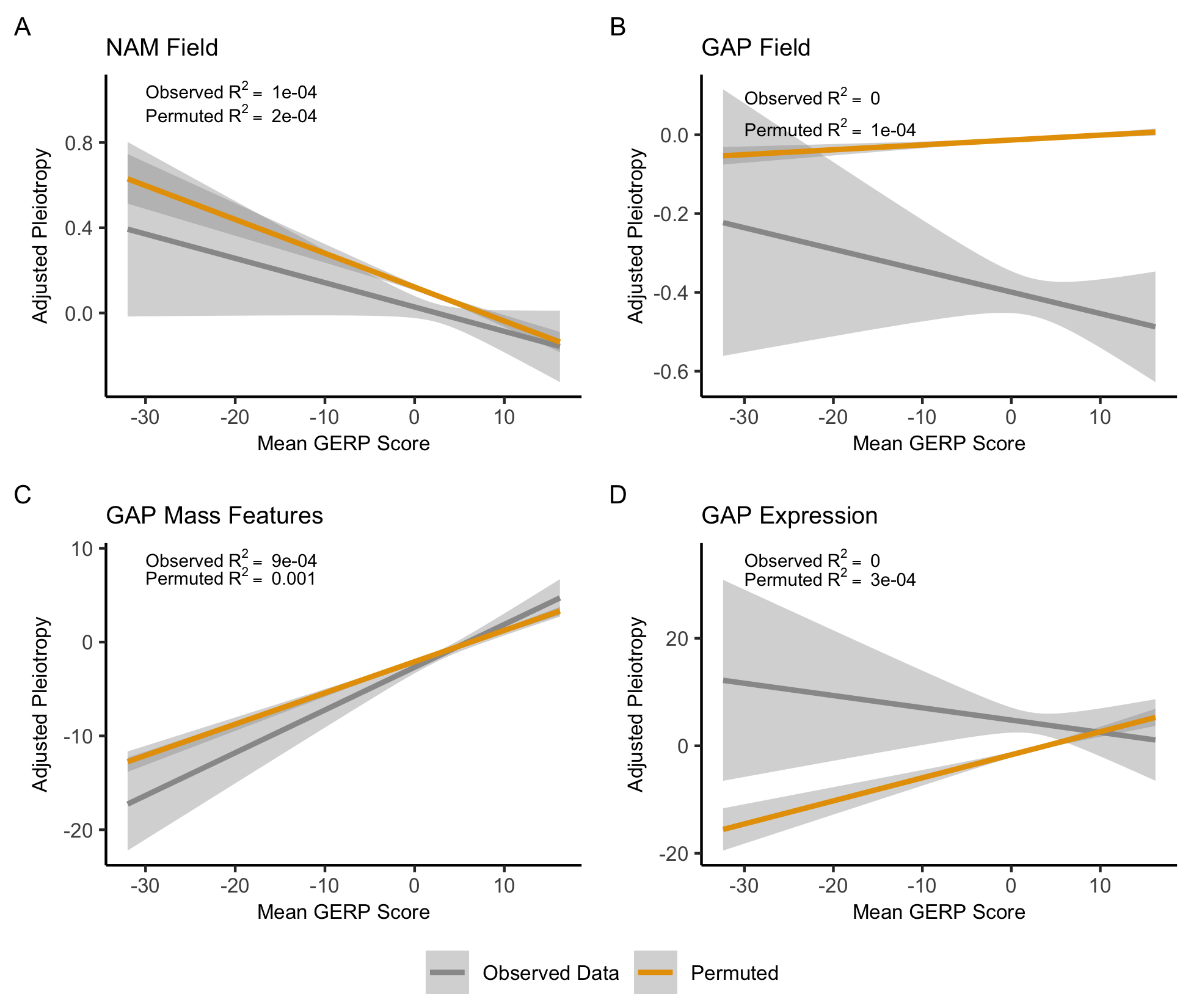

Supplement: S19 Fig — Line plots show adjusted pleiotropy against the mean GERP score for all genic and intergenic intervals. Mean GERP was calculated by averaging overlapping observed GWA SNPs with GERP SNPs for each population-trait category. Results for the observed data are in gray, while permuted results are in yellow. Panels show adjusted pleiotropy scores for (a) NAM field, (b) GAP field, (c) GAP mass features, and (d) GAP expression traits. Values in the top right of each plot show the R2 values from correlating the observed and permuted adjusted pleiotropy data against the mean GERP score. Shading around lines shows the 95% confidence interval. (TIF) [file pgen.1010664.s028.tif]

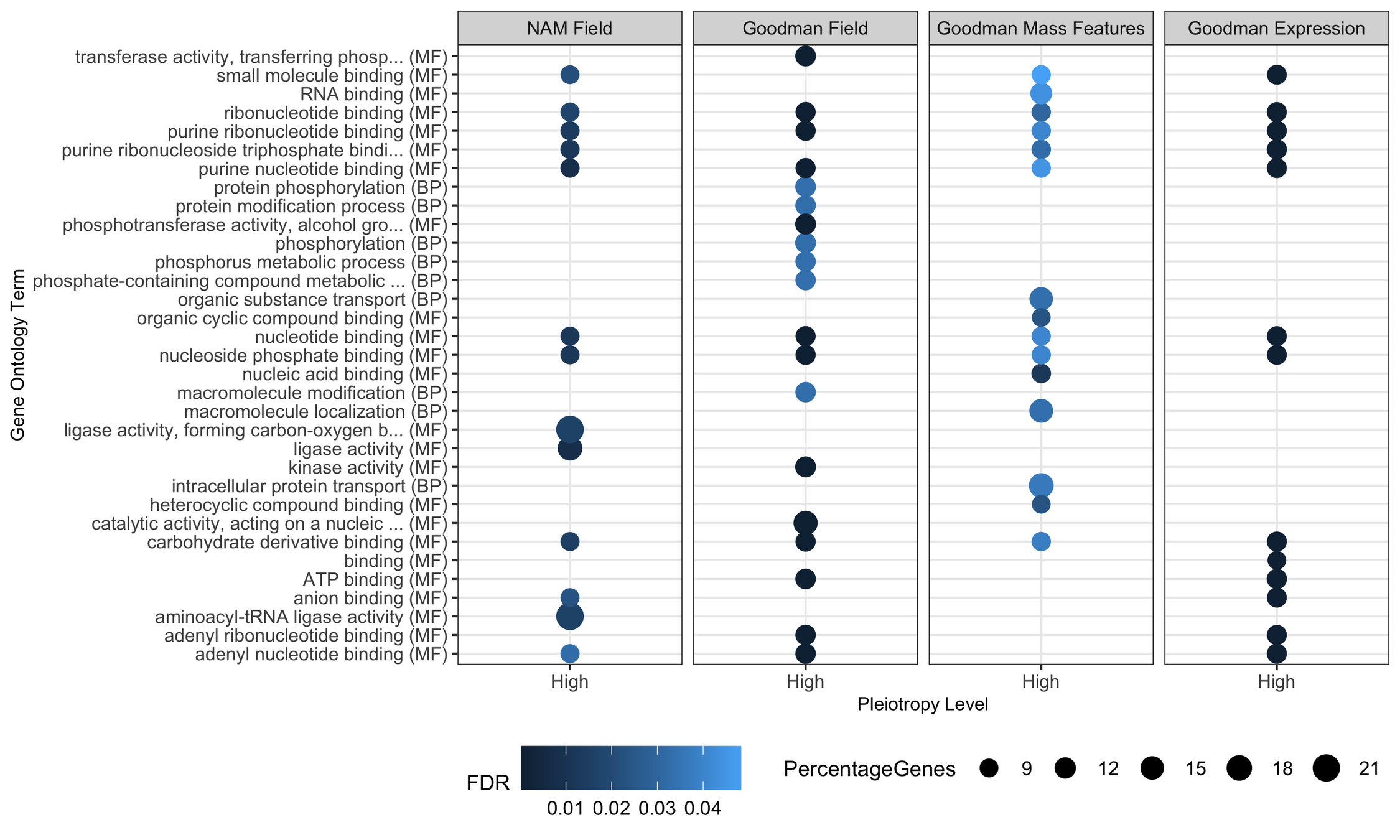

Supplement: S20 Fig — All molecular function, biological process, and cellular component terms were tested. Terms with FDR corrected p-values below p-value < 0.05 were retained. The x-axis represents highly or lowly pleiotropic intervals split by the population and trait type, and the y-axis shows the top significant gene ontology terms. The value of the FDR significance level is colored in blue and the size of the dots represents the proportion of genes found in that GO category. (TIF) [file pgen.1010664.s029.tif]

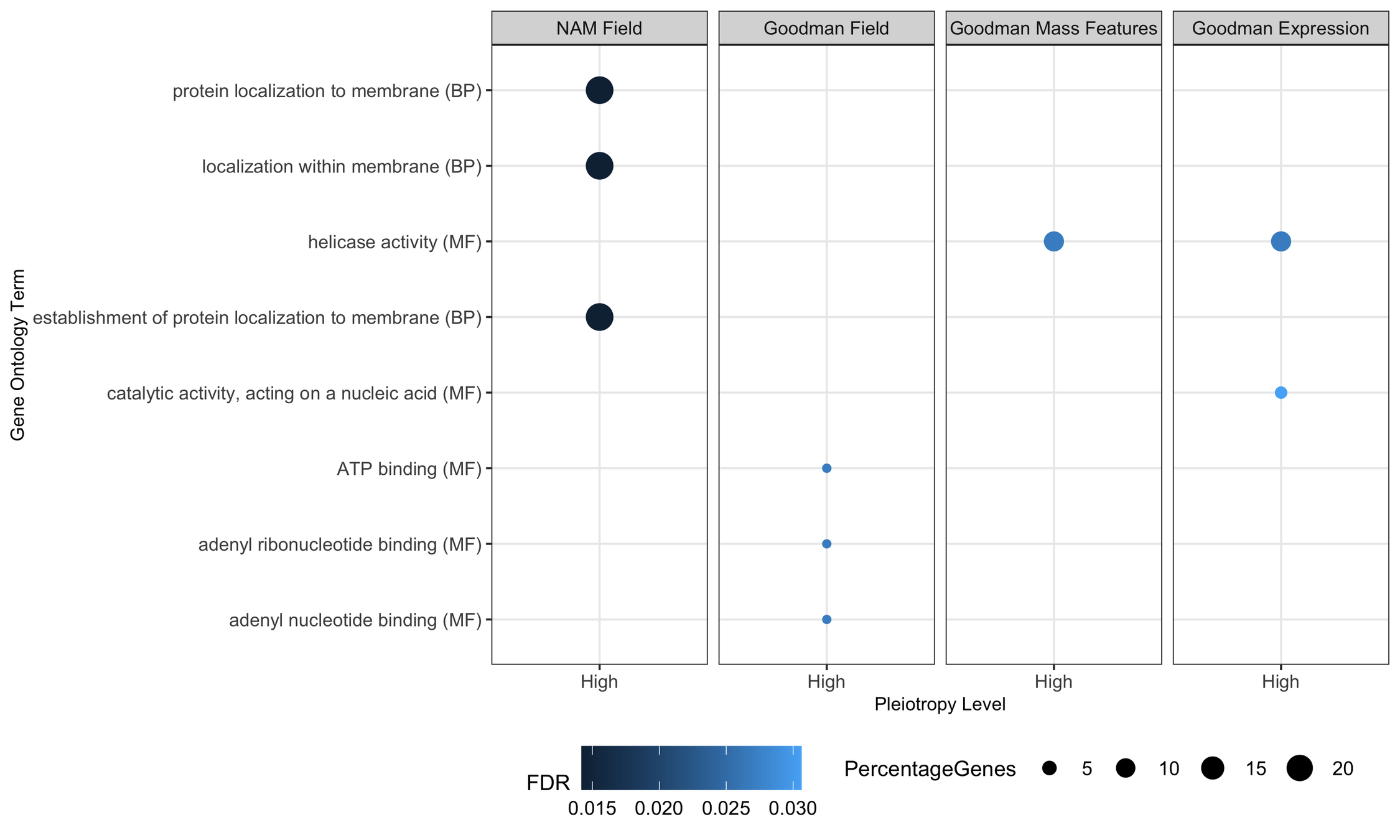

Supplement: S21 Fig — All molecular function, biological process, and cellular component terms were tested. Terms with FDR corrected p-values below p-value < 0.05 were retained. The x-axis represents highly or lowly pleiotropic intervals split by the population and trait type, and the y-axis shows the top significant gene ontology terms. The value of the FDR significance level is colored in blue and the size of the dots represents the proportion of genes found in that GO category. (TIF) [file pgen.1010664.s030.tif]
